# Isotopic signatures and source apportionment of Pb in ambient PM_2.5_

**DOI:** 10.1038/s41598-022-08096-1

**Published:** 2022-03-14

**Authors:** Chien-Cheng Jung, Charles C.-K. Chou, Yi-Tang Huang, Shih-Yu Chang, Chung-Te Lee, Chuan-Yao Lin, Hing-Cho Cheung, Wei-Chen Kuo, Chih-Wei Chang, Shuenn-Chin Chang

**Affiliations:** 1 Department of Public Health, China Medical University, Taichung, Taiwan; 2 Research Center for Environmental Changes, Academia Sinica, Taipei, Taiwan; 3 Department of Public Health, Chung Shan Medical University, Taichung, Taiwan; 4 Graduate Institute of Environmental Engineering, National Central University, Taoyuan, Taiwan; 5 Meteorological Research Institute (MRI), Tsukuba, Japan; 6 Environmental Protection Administration, Taipei, Taiwan; 7 School of Public Health, National Defense Medical Center, Taipei, Taiwan

**Keywords:** Environmental chemistry, Environmental impact

## Abstract

Particulate lead (Pb) is a primary air pollutant that affects society because of its health impacts. This study investigates the source sectors of Pb associated with ambient fine particulate matter (PM_2.5_) over central-western Taiwan (CWT) with new constraints on the Pb-isotopic composition. We demonstrate that the contribution of coal-fired facilities is overwhelming, which is estimated to reach 35 ± 16% in the summertime and is enhanced to 57 ± 24% during the winter monsoon seasons. Moreover, fossil-fuel vehicles remain a major source of atmospheric Pb, which accounts for 12 ± 5%, despite the current absence of a leaded gasoline supply. Significant seasonal and geographical variations in the Pb-isotopic composition are revealed, which suggest that the impact of East Asian (EA) pollution outflows is important in north CWT and drastically declines toward the south. We estimate the average contribution of EA outflows as accounting for 35 ± 15% (3.6 ± 1.5 ng/m^3^) of the atmospheric Pb loading in CWT during the winter monsoon seasons.

## Introduction

Scientific studies have demonstrated that exposure to lead (Pb) particles is associated with hypertension^1,2^. It has also been indicated that a Pb level higher than 10 mg/dL in blood could result in a decline in the birth weight of infants^3^ and intellectual impairment in children^4^. Studies have found that even low-level Pb exposure could cause damage to the nervous system^5,6^ and hippocampus^7,8^ and increase the risk of cognitive dysfunction^9^. The World Health Organization^10^ recently published a report and indicated that Pb exposure was linked to irreversible neurological damage and caused 0.90 million deaths in 2019.

In the 1970s, Pb-alkyl additives in gasoline were a major source of Pb in the atmosphere^11,12^. Given the scientific evidence of the health impacts of lead exposure, the use of leaded fuels had been banned by some national governments in the 1980s and a worldwide phase-out was just announced by the United Nations Environment Programme (UNEP) recently. Consequently, a notable reduction in the ambient Pb level was achieved. For example, a study using reservoir sediment cores showed that the Pb level sharply declined from 1500 ng/m^3^ in 1975 to 70 ng/m^3^ in 1990 in the US^13^, and some studies found decreases in ambient Pb level from 700 ng/m^3^ in 1991 to 2.6 ng/m^3^ in 2015 in Taipei, Taiwan^14–16^. However, elevated Pb levels have still been observed in recent studies in Taiwan, US, Czech, Poland, Saudi Arabia, and China, suggesting substantial air quality impacts of other sources^14,15,17–21^. Therefore, Pb remains a critical public health concern^22^. Investigations on the sources of Pb-containing particles have therefore been conducted, which have reported emissions of Pb stemming from a wide range of sources including but not limited to road dust, incinerators, coal-fired power plants, mines, bedrock, and construction work^23–29^.

Because Pb-containing particles originating from various sources usually occur mixed in ambient fine particulate matter (PM_2.5_), the identification and apportionment of sources of Pb particles is a highly challenging issue in air quality management. The positive matrix factorization (PMF) model is a statistical tool that is widely applied in air pollution investigations^14,29–31^ and can resolve the contribution matrix of particulate matter pollution sources according to profiles of the chemical composition. However, the attribution of each factor to a specific pollution source is highly uncertain because the chemical profiles of pollution sources may exhibit common features^32^. Recently, scientists started to explore the isotopic characteristics of Pb collected from specific sources or ambient air^33–36^ and, in turn, have assessed the impacts of various sources^15,27,37,38^. Studies have determined that the Pb isotopic composition changes due to variations in oil consumption and unleaded gasoline usage^39–42^ and have suggested the major role of industrial emissions in present-day Pb pollution.

Taiwan is located offshore of southeastern China and is thus subject to the impacts of air pollution associated with East Asian (EA) continental outflows during the winter monsoon seasons. In addition, western Taiwan is a highly developed area with a population of approximately 23 million people and a large number of industrial factories, which emit a substantial amount of air pollutants. In this study, we present an in-depth analysis on two datasets, which were produced by the Taiwan EPA PM_2.5_ speciation program in 2017–2019 and an intensive investigation conducted in the central-western Taiwan (CWT) from 2016 to 2018, respectively. Details of the two datasets are described in the section of Dataset and Method. We include the isotopic composition of Pb in the chemical profile of PM_2.5_ samples and the Pb isotopic features of each pollution factor are then resolved with the PMF model, which adds new dimensions in source apportionment and helps to attribute Pb pollution to specific sources. Moreover, analysis of the geographical distribution of the Pb isotope ratios (i.e., ^206^Pb/^207^Pb and ^208^Pb/^207^Pb) of PM_2.5_ samples is conducted to investigate the influences of air pollution originating from local sources and/or transported by EA continental outflows during the winter monsoon seasons.

## Results

### Ambient concentration and mass mixing ratio of Pb in PM_2.5_

This subsection presents analysis of the Pb content in the samples collected under the PM_2.5_ speciation program of the Taiwan Environmental Protection Administration (EPA) during the period from 2017–2019. The average ambient concentration of Pb in PM_2.5_ reached 7.9 ± 8.2, 8.3 ± 7.4 and 16.0 ± 22.1 ng/m^3^ at the Zhongming (ZM), Douliu (DL), and Chiayi (CY) stations, respectively. The geographical locations of these sampling sites are shown in Supplementary Fig. 1. The results indicate that the Pb concentrations at Zhongming and Douliu are highly correlated (r = 0.7386), whereas the Pb concentration at Chiayi exhibits significantly different features (p < 0.01). In contrast, the corresponding PM_2.5_ levels were 21.1 ± 11.5, 26.3 ± 14.3, and 26.5 ± 15.0 μg/m^3^ at the three stations from 2017–2019. The PM_2.5_ collected at Chiayi is characterized by an average Pb mass mixing ratio of 533 ± 613 ppm, which is 51% and 85% higher than the ratios of 354 ± 271 ppm and 289 ± 178 ppm, reported at the Zhongming and Douliu stations, respectively.

**Figure 1:**
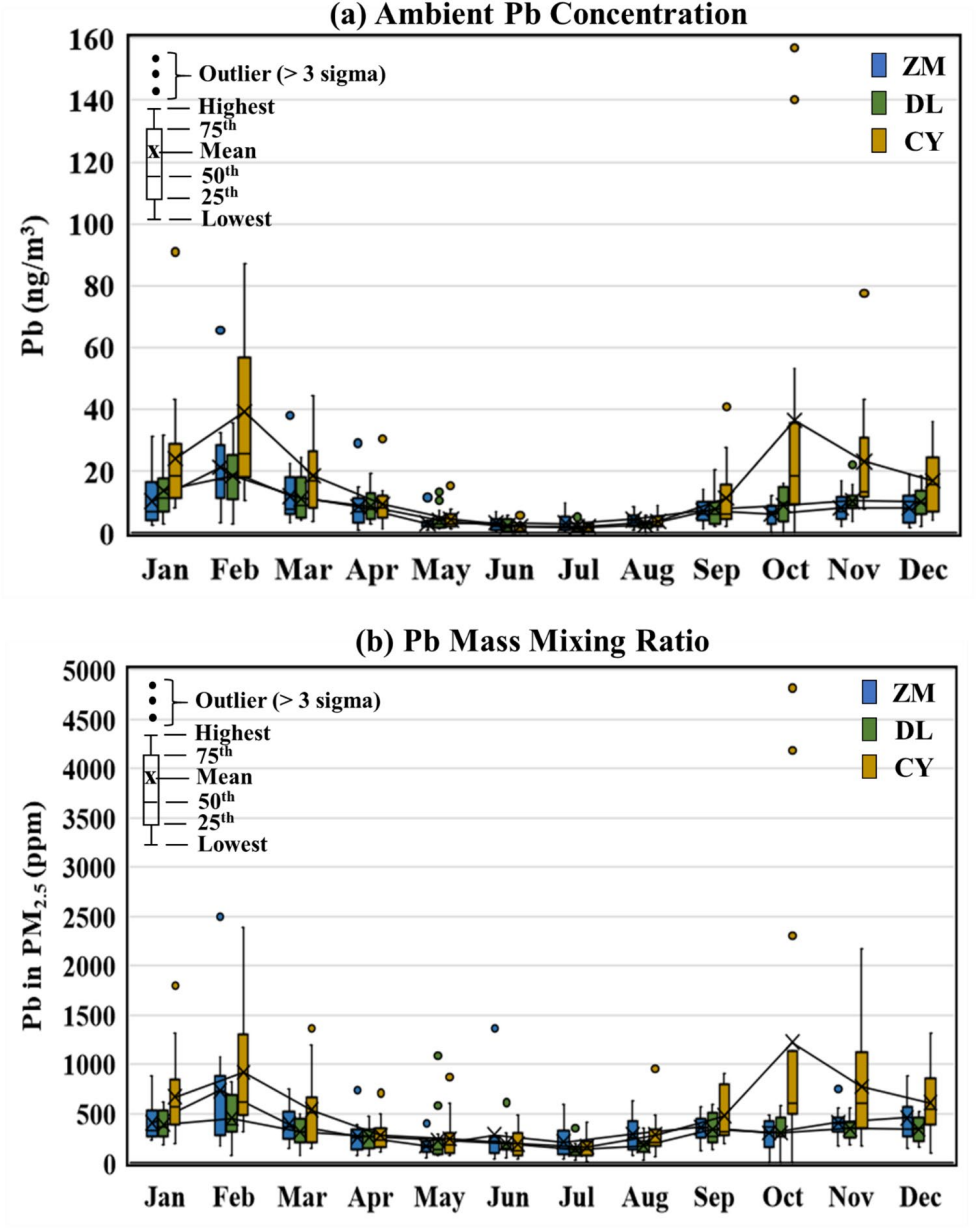
Seasonal variations in the ambient concentrations (**a**) and mass mixing ratios (**b**) of Pb in PM_2.5_. Samples are collected at the Zhongming (ZM), Douliu (DL), and Chiayi (CY) sites of the Taiwan EPA PM_2.5_ speciation network from 2017–2019. Geographic information on the sampling sites is shown in Supplementary Fig. 1.

Figure 1 shows that both the ambient concentration and mass mixing ratio of Pb in PM_2.5_ are associated with significant seasonal variations. The decline in the ambient Pb concentration in summer agrees with the typical air pollution pattern, which is usually the result of atmospheric dispersion enhancement^14,15^. However, the amplitude of the seasonal variation in the Pb concentration (i.e., winter mean/summer mean) ranges from 3.8–10.2 at the three sites, which is significantly larger than the amplitude of the variation in PM_2.5_. The seasonal variation in the mass mixing ratio of Pb in PM_2.5_ suggests that Pb more abundantly occurs in PM_2.5_ during the period from September to April of the next year, which indicates that the sources of PM_2.5_ could have changed with the seasons in CWT, and thereby merit an in-depth investigation.

### Isotopic composition of Pb in PM_2.5_

In order to facilitate investigation on the seasonal changes in the sources of Pb-containing particles in the CWT, Table 1 summarizes the isotopic composition of Pb in the PM_2.5_ samples collected during the intensive investigation that was performed from 2016 to 2018. The overall average PM_2.5_ concentrations was 23.5 ± 13.7 μg/m^3^ in the study area, ranging from 20.0 ± 13.0 to 25.9 ± 17.0 μg/m^3^ at the respective sites. The overall average ambient Pb concentration in PM_2.5_ was 8.6 ± 7.2 ng/m^3^, with the site-specific average concentrations ranging from 3.3 ± 2.2 to 15.6 ± 12.7 ng/m^3^. While the overall average ambient Pb level during the sampling campaigns is comparable to the year round averages that reported in the previous subsection, this investigation reveals pronounced spatial differences in the ambient Pb concentration.

**Table 1:** Summary of the average concentrations of PM_2.5_ and Pb associated with Pb-isotopic composition during the intensive field investigation.

Sampling site	PM_2.5_ (μg/m^3^)	Pb (ng/m^3^)	^206^Pb/^207^Pb	^208^Pb/^207^Pb
Changhua (CH)	22.6 ± 12.1	9.6 ± 7.1	1.149 ± 0.008	2.429 ± 0.012
Douliu (DL)	24.8 ± 15.4	9.3 ± 7.0	1.145 ± 0.011	2.426 ± 0.015
Dali (DA)	25.0 ± 11.8	5.0 ± 3.9	1.147 ± 0.008	2.426 ± 0.015
Fengyuan (FY)	23.1 ± 9.6	3.7 ± 1.6	1.151 ± 0.010	2.429 ± 0.013
Chiayi (CY)	25.9 ± 17.0	15.6 ± 12.7	1.146 ± 0.008	2.421 ± 0.007
Erlin (EL)	23.7 ± 14.5	10.1 ± 6.5	1.153 ± 0.008	2.430 ± 0.011
Shalu (SL)	24.4 ± 12.5	3.3 ± 2.2	1.137 ± 0.011	2.417 ± 0.013
Xianxi (XX)	24.2 ± 11.7	6.1 ± 4.1	1.152 ± 0.008	2.424 ± 0.010
Xingang (XG)	24.7 ± 15.9	10.1 ± 6.3	1.147 ± 0.008	2.429 ± 0.008
Zhushan (ZS)	22.2 ± 13.5	7.1 ± 5.9	1.146 ± 0.008	2.429 ± 0.010
Mailiao (ML)	21.8 ± 14.1	8.0 ± 5.7	1.149 ± 0.010	2.424 ± 0.013
Lunbei (LB)	25.0 ± 15.7	9.9 ± 6.1	1.149 ± 0.008	2.428 ± 0.013
Taixi (TX)	20.0 ± 13.0	6.9 ± 5.1	1.153 ± 0.010	2.432 ± 0.016
Overall	23.5 ± 13.7	8.6 ± 7.2	1.148 ± 0.009	2.427 ± 0.012

**Figure 2:**
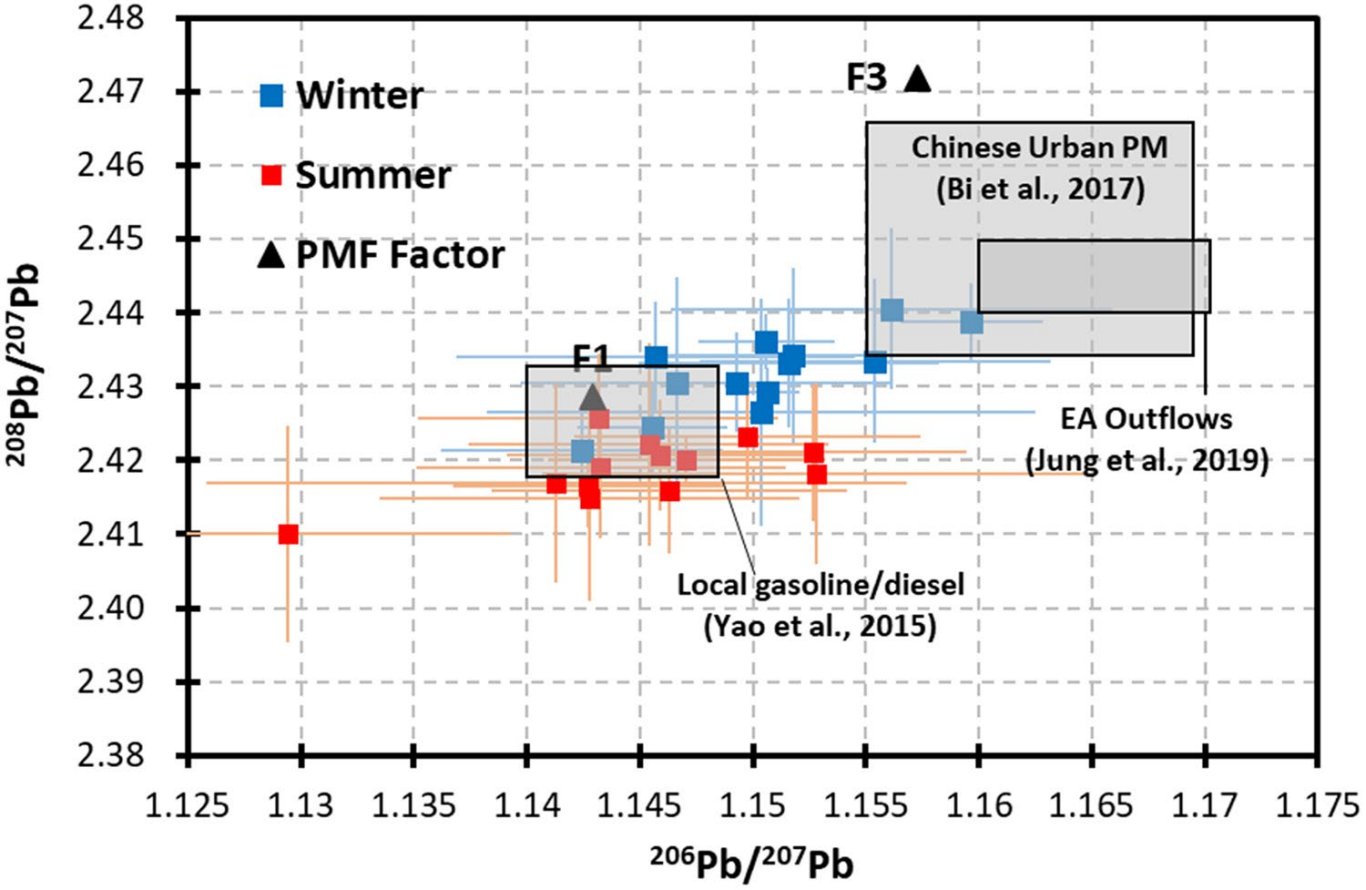
Distribution of the isotopic ratios of Pb in PM_2.5_ in the summer and winter samples. Samples are collected at 13 sampling sites in central-western Taiwan during 5 field campaigns from 2016–2018, as described in the main text. The site-specific averages are denoted by symbols with ranges of ± 1 standard deviation, as shown by the error bars. The isotopic ratios of 2 PMF factors (F1 and F3) are denoted by triangles, whereas the other factors are beyond the ranges of this plot. The ranges of the isotopic ratios for fuels supplied in Taiwan, Chinese urban particulate matter and PM_2.5_ transported by EA outflows are indicated with gray squares, with relevant references noted.

The overall average values of the ^206^Pb/^207^Pb and ^208^Pb/^207^Pb ratios were 1.148 ± 0.009 and 2.427 ± 0.012, respectively. Considering the potential seasonal changes in the sources of Pb in PM_2.5_, the sampling campaigns were further divided into summer (i.e., August 2017 and July 2018) and winter (November 2016, February 2017 and March 2018) campaigns. The summer average values of ^206^Pb/^207^Pb and ^208^Pb/^207^Pb were 1.145 ± 0.010 and 2.419 ± 0.011, respectively, whereas the winter average values reached 1.150 ± 0.008 and 2.432 ± 0.011, respectively. Both isotopic ratios exhibited significant seasonal differences (p < 0.01). Figure 2 illustrates the correlation between ^206^Pb/^207^Pb and ^208^Pb/^207^Pb. The two isotopic ratios maintained a significant linear correlation, whereas a steeper slope was associated with the winter dataset. A larger slope value indicates a significant increase in the relative abundance of ^208^Pb during the winter monsoon seasons. Moreover, it should be noted that both ^206^Pb/^207^Pb and ^208^Pb/^207^Pb exhibited an increasing trend and moved toward the case representing EA continental outflows (as shown in Fig. 2) during the winter monsoon seasons.

### Source identification of Pb in PM_2.5_

To resolve the contribution of the pollution sources responsible for Pb in PM_2.5_, this study employed the PMF model to analyze the data obtained during the intensive investigation. Note that the 3 Pb isotopes (^206^Pb, ^207^Pb, and ^208^Pb) were independently considered in the chemical profiles, and the retrieved factors were in turn characterized based on Pb isotopic ratios (i.e., ^206^Pb/^207^Pb and ^208^Pb/^207^Pb). PMF analysis provided a solution involving 8 factors. The chemical profiles of respective factors are illustrated in Supplementary Fig. 2. However, considering the uncertainties associated with the measurements and source apportionment, we examined only the top 4 factors in this study. Attribution of the source factors was based on the characteristic elements of the chemical profiles. In summary, the four major source factors of Pb included the following: (1) traffic emissions, (2) the petrol industry, (3) coal-fired facilities, and (4) oil-fired facilities. Notably, all four major sources were related to the production and/or use of fossil fuels. Table 2 summarizes the contribution of 4 major source factors to the total Pb in PM_2.5_ in the study area and the Pb isotopic ratios and characteristic elements considered to achieve source attribution, whereas a summary of all the 8 factors is provided in Supplementary Table 3. In total, the top 4 factors accounted for ~ 80% of the total Pb in PM_2.5_. Note that the above attribution of source factors resolved by the PMF model was based simply on the characteristic elements illustrated in the chemical profiles, but there could be contributions of unidentified minor sources not accounted for.

**Table 2:** Summary of source apportionment for Pb in PM_2.5_ and Pb isotopic composition of the respective source factors resolved by the PMF model.

PMF factor (attribution)	Contribution, %*	^206^Pb/^207^Pb	^208^Pb/^207^Pb	Characteristic constituents^#^
F1 (traffic emissions)	12 ± 5 (S: 12 ± 6 W: 12 ± 7)	1.146 ± 0.005	2.418 ± 0.009	Zn (51%), Cu (31%), Mn (23%), Pb (12%) (Lin et al.^26^)
F2 (petrol industry)	11 ± 4 (S: 16 ± 8 W: 8 ± 3)	1.148 ± 0.009	2.292 ± 0.008	Nd (56%), Ce (51%), Ti (48%), La (39%), Sr (26%) (Chow^55^; Kulkarni et al.^56^; Moreno et al.^57^)
F3 (coal-fired facilities)	49 ± 12 (S: 35 ± 16 W: 57 ± 24)	1.159 ± 0.004	2.467 ± 0.006	Cd (57%), Pb (52%), As (40%), Se (32%) (Okuda et al.^58^)
F4 (oil-fired facilities)	10 ± 5 (S: 21 ± 8 W: 6 ± 2)	1.126 ± 0.004	2.278 ± 0.005	V (65%), Ni (43%) (Querol et al.^59^; Cheng et al.^60^)

*Averages for summer (S) and winter (W) campaigns are listed in parentheses.

^#^The percentage of variations in the characteristic elements attributed to each PMF factor is listed in parentheses.

The contribution of traffic emissions (Factor 1) to Pb in PM_2.5_ was estimated to reach 12 ± 5% in the study area despite the current lack of leaded gasoline usage. The attribution of this factor was based not only on the dominance of the variations in Cu, Mn and Zn in PM_2.5_ but also on the Pb isotopic ratios. This factor was characterized by ^206^Pb/^207^Pb and ^208^Pb/^207^Pb ratios of 1.146 and 2.418, respectively. As shown in Fig. 2, the Pb isotopic features are consistent with the features of gasoline and diesel supplied in Taiwan^43^.

The second source factor was attributed to the petrol industry due to the abundance of source-specific characteristic elements, in particular La, Ce, and Nd^44^. The contribution of this factor was estimated to reach 11 ± 4% throughout the entire study period, whereas a higher contribution (16 ± 8%) was observed in summer. This is justified by the geographic location of local sources in the study area. Several petroleum refining factories are located in southern Taiwan, which occurs upwind of our study sites during the summer monsoon seasons.

Coal-fired facilities (Factor 3) contributed almost half (49 ± 12%) of Pb in PM_2.5_ in this area. This source factor exhibited significant seasonal differences, contributing 35 ± 16% to the Pb loading during the summer campaigns and 57 ± 24% during the winter campaigns on average. This factor was characterized by ^206^Pb/^207^Pb and ^208^Pb/^207^Pb ratios of 1.159 and 2.467, respectively. As shown in Fig. 2, this Pb isotopic feature is similar to that of aerosol samples collected in Chinese urban areas with significant pollution attributed to coal combustion^41^. It has been previously reported that the concentration and mixing ratio of Pb in PM_2.5_ are enhanced during the EA winter monsoon seasons, suggesting influences of pollution transported from eastern and/or northern China. Here, the argument is further supported by Pb isotope evidence.

The 4th source factor encompassed oil-fired facilities because the variations in Ni and V in PM_2.5_ were predominant. Given that residue oil is used mostly in heavy duty engines and boilers, this source was likely related to the emissions of ships and industrial facilities. This factor contributed 10 ± 5% to the Pb loading throughout the entire study period, whereas a significantly high contribution (21 ± 8%) was estimated during the summer campaigns. This seasonality was further justified by the geographic distribution of pollution sources in Taiwan. The majority of the heavy industry is located in southern Taiwan. Moreover, this factor could have been influenced by local circulation because sea breezes prevail in summer and thus could transport more ship emissions from the Taiwan Strait into the study area.

## Discussion

### Pb isotopic fingerprints of coal-fired facilities

The results in this study indicate that the ambient concentration and mass mixing ratio of Pb in PM_2.5_ over CWT were significantly higher during the EA winter monsoon seasons than those during the summertime. Analysis of the back-trajectories of the air masses arriving in the study area on the sampling days reveals that Taiwan was influenced by EA outflows during the winter campaign periods and by southwesterlies during the summer campaign periods (as shown in Supplementary Fig. 4). PMF analysis determined the predominant factor of the contribution of coal-fired facilities (F3), which accounted for 57 ± 24% of the ambient Pb loading during the wintertime. Accordingly, it is plausible that the emission of Pb-containing particles by coal-fired facilities in China is a predominant source of atmospheric Pb in the CWT area during the wintertime. However, PMF analysis also determined a substantial contribution (35 ± 16%) of this factor (F3) during the summer campaigns, when Taiwan is typically impacted by southwesterly monsoons and isolated from the influences of Chinese air pollution. Thus, there could occur local coal-fired facilities possessing certain Pb isotope fingerprints, such as that of F3 (^206^Pb/^207^Pb: 1.159 ± 0.004, ^208^Pb/^207^Pb: 2.467 ± 0.006). Within this context, an investigation of the Pb isotopic features of the coal used by local suspected facilities is warranted to identify the sources of atmospheric Pb in Taiwan.

### Geographical distribution of the isotopic composition of Pb in PM_2.5_

**Figure 3:**
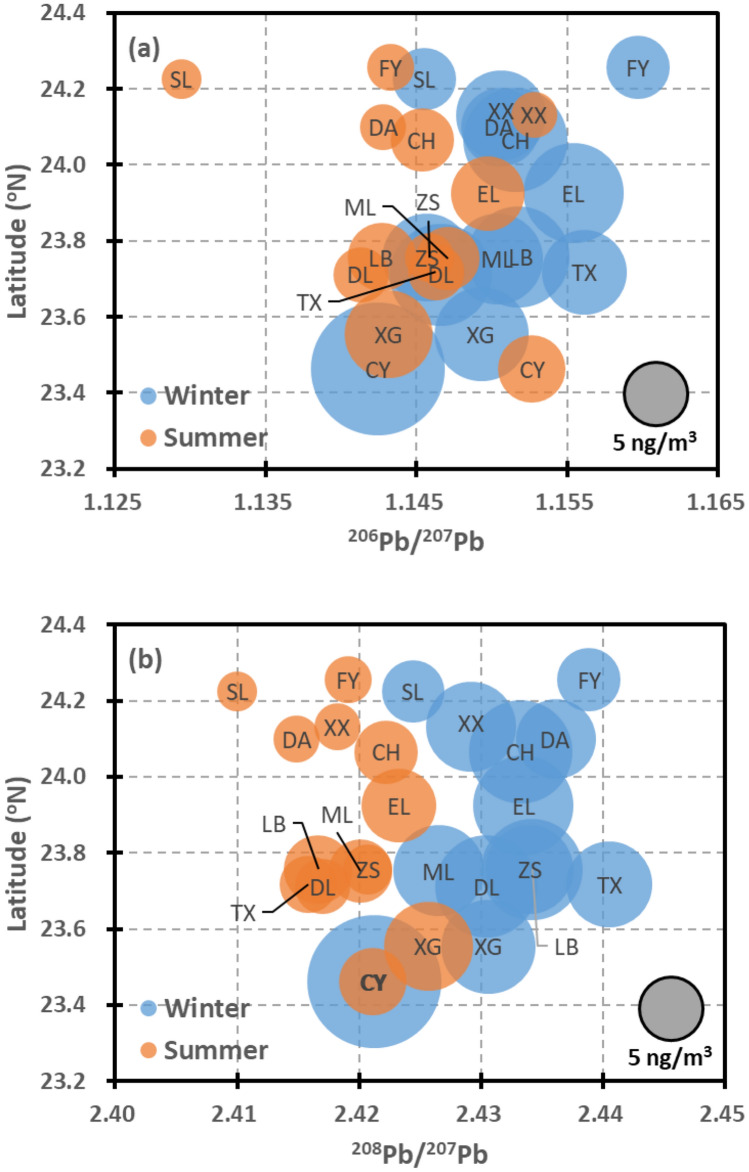
Geographical distribution of the ambient Pb levels and isotopic ratios, ^206^Pb/^207^Pb (**a**) and ^208^Pb/^207^Pb (**b**), along latitudes during the winter and summer campaigns. The circle area is proportional to the ambient mass concentration of Pb in PM_2.5_. The coordinates of the respective sampling sites are listed in Supplementary Table 1, and labels of respective sites are **CH:** Changhua; **CY:** Chiayi; **DA:** Dali; **DL:** Douliu; **EL:** Erlin; **FY:** Fengyuan; **LB:** Lunbei; **ML:** Mailiao; **SL:** Shalu; **TX:** Taixi; **XG**: Xiangang; **XX:** Xianxi; **ZS:** Zhushan.

This study reveals significant spatial differences in the ambient Pb level among the sampling sites. Based on the previous section, the PM_2.5_ samples collected at the Chiayi site are more enriched in Pb than those collected at the other sites. The results of our intensive investigation (as listed in Table 1) confirm the high Pb level at Chiayi and indicate lower Pb levels at the Fengyuan and Shalu sites. Figure 3 shows the changes in the average ambient Pb level and isotopic ratios at each sampling site with the latitude. It is apparent that the ambient Pb level increases from the northern to the southern parts of the study area, where Fengyuan and Chiayi are located at the northern and southern ends, respectively. The average Pb isotopic ratios (^206^Pb/^207^Pb and ^208^Pb/^207^Pb) of PM_2.5_ at Chiayi are 1.142 ± 0.006 and 2.421 ± 0.006, respectively, and 1.153 ± 0.007 and 2.421 ± 0.009, respectively, during the winter and summer campaigns, respectively. In contrast to the general seasonal shift in the Pb isotopic ratios presented above and shown in Fig. 3b, the ^208^Pb/^207^Pb ratio at Chiayi does not significantly vary with the season. Given the high ^208^Pb/^207^Pb values associated with Chinese air pollutants, as shown in Fig. 2, the isotopic data suggest that the influences of EA outflows on the Pb level at Chiayi are relatively minor. In contrast, the average Pb isotopic ratios (^206^Pb/^207^Pb and ^208^Pb/^207^Pb) of PM_2.5_ at Fengyuan during the winter campaigns are 1.160 ± 0.003 and 2.439 ± 0.005, respectively, which are comparable to the characteristics of the aerosols transported by EA outflows, as shown in Fig. 2. As a result, it is inferred that Fengyuan is significantly influenced by EA outflows during the EA winter monsoon seasons. Notably, the summer-campaign average values of the ^206^Pb/^207^Pb and ^208^Pb/^207^Pb ratios of PM_2.5_ at Fengyuan shift to 1.143 ± 0.008 and 2.419 ± 0.010, respectively, which are consistent with the winter Pb isotopic features of PM_2.5_ at Chiayi. These seasonal and geographical shifts in the Pb isotopic features suggest variations in the transport of air pollutants, which will be further elaborated in the following section.

### Estimation of the contribution of EA outflows to local Pb loadings

Given the narrow range of ^208^Pb/^207^Pb during the summertime and the significant spatial and seasonal shifts, as shown in Fig. 3b, we employ a two-end-member model to estimate the contribution of EA outflows during the winter campaigns in this study. We assume that the overall average ^208^Pb/^207^Pb value over the summer campaigns, i.e., 2.419 ± 0.011, represents the features of local pollution, while the average ^208^Pb/^207^Pb value based on Chinese urban measurements, as reported by Bi et al.^41^, namely, 2.453 ± 0.009, represents the end member of EA outflows. Note that only the investigations conducted after 2000 are included here, considering the changes in pollution sources across China. As a result, it is estimated that EA outflows contribute 35 ± 15% or 3.6 ± 1.5 ng/m^3^ to the ambient Pb loading over CWT during the winter monsoon seasons. The site-specific estimates are summarized in Supplementary Table 2. It should be noted that the influences of EA outflows are more pronounced along the western coastline and exhibit a declining trend toward the southern and inland (eastern) areas. There are two exceptional cases, namely, Shalu and Mailiao, where the PM_2.5_ properties could have been dominated by specific sources located near these sampling sites.

**Figure 4:**
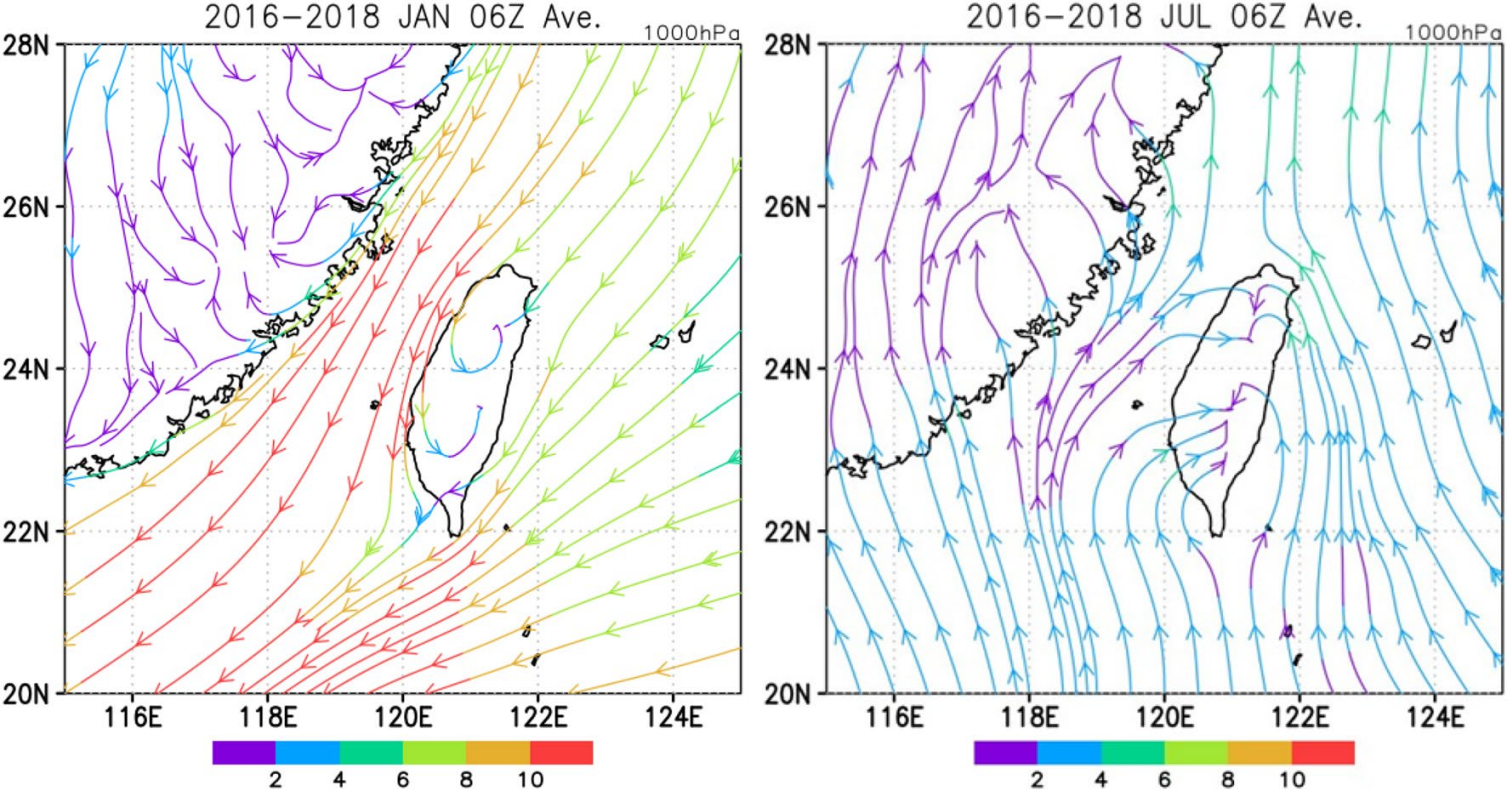
Mean surface streamlines in winter (January) and summer (July) from 2016–2018. The northeasterly East Asian winter monsoon branches reveal geographic blocking over Taiwan Island, which allows the development of side flows moving toward southeastern Taiwan. Weak southerly flows prevail during the summertime, and sea breezes dominate the transport of local air pollutants. The meteorological data at 06 UTC are used to demonstrate the general pattern of the wind field. The data are retrieved from the Global Data Assimilation System (GDAS).

Within the context of monsoon activity and the Pb isotopic features of PM_2.5_ described above, the results in this study demonstrate that the influences of EA outflows could have drastically declined within only ~ 1 degree in latitude, i.e., from Fengyuan to Chiayi. This is explained by the development of local circulation in CWT. Figure 4 illustrates the mean surface (1000 hPa) streamlines around Taiwan calculated for January and July from 2016–2018, which represent the general transport patterns of air parcels in winter and summer, respectively. The streamlines indicate that the main stream of EA outflow is geographically blocked by the Central Mountain Ranges of Taiwan. As a result, a side flow moving toward southeastern Taiwan develops, particularly during the daytime. Consequently, the impacts of EA outflows diminish, and the pollutants emitted in the coastal areas of Taiwan could have dominated the air quality in the southern part of the study area. In contrast, the regional wind field is dominated by slow southerly flows during the summertime, and strong sea breezes could have driven local air pollutants eastward. The seasonal changes in local circulation suitably elucidate the geographical shift in the isotopic composition of Pb in PM_2.5_.

## Dataset and method

### Datasets

Two datasets of Pb in PM_2.5_ were analyzed and presented in this study. The first dataset is on the mass concentration of Pb measured at three sampling sites of Taiwan EPA during 2017–2019. The Taiwan EPA’s sampling network comprises six stations distributed across Taiwan, and three of these six sites (Zhangming, Douliu, and Chiayi) are located within our study area. Geographic locations of the sampling sites are illustrated in the Supplementary Fig. 1. Under this program, each sample was collected over a 24-h period, and sampling was conducted regularly at 6-day intervals throughout the year. Thus, the advantage of this dataset is that it contains measurement for a whole year; it hence provides a more representative data of the ambient Pb level in the study area and a better picture for the spatial and seasonal variations in the ambient Pb concentration. Unfortunately, this program was initiated in 2017, and did not provide data for 2016. Moreover, the sampling sites were relatively sparse: only 3 sites located in our study area. The second dataset is the major dataset of this study, which is on the detailed chemical characterization of PM_2.5_ samples collected from 13 sites during 5 sampling campaigns in 2016–2018, which is described in further details in the following. Geographic locations of the sampling sites are also illustrated in the Supplementary Fig. 1. The major advantage of this dataset is that it provides isotopic measurement of Pb for each PM_2.5_ sample. However, because of the limited capacity of sampling/analysis in lab, the sampling experiments were designed to investigate the winter/summer features and, thereby, did not provide a year-round seasonal variation. Moreover, we put more efforts to the spatial distribution and, thereby, only a limited number of samples were collected at each site.

### Sample collection and chemical analysis of intensive investigation

An intensive investigation on the attribution of Pb in PM_2.5_ was conducted in CWT from 2016–2018. A total of 274 daily PM_2.5_ samples were collected at 13 sites distributed among urban, rural, and industrial zones across the study area during 5 sampling campaigns. PM_2.5_ samples were simultaneously collected at the sites on a daily base for 7 to 10 days during each sampling campaign. Detailed information on the site categorization and sampling campaigns is provided in Supplementary Table 1.

At each sampling site, a BGI PQ200 sampler with airflow at 16.7 L/min (Mesa Labs, Inc., Butler, NJ, USA) was employed to collect PM_2.5_ on Teflon filters, which were used for gravimetric measurement and analysis of crustal and metal elements (Al, As, Ca, Cd, Ce, Co, Cr, Cs, Cu, Fe, Ge, La, Mn, Mo, Nd, Ni, Pb, Rb, Sb, Se, Sn, Sr, Ti, Tl, V, Zn, Zr) and Pb isotopes (^206^Pb, ^207^Pb, and ^208^Pb). In addition, a multichannel Super-SASS sampler with airflow at 6.7 L/min at each channel (Met One Instruments Inc., Grants Pass, OR, USA) was deployed to collect PM_2.5_ on Teflon and tissue quartz-fiber filters. The Teflon filter samples were reserved for the analysis of soluble ions (Na^+^, NH_4_^+^, K^+^, Mg^2+^, Ca^2+^, Cl^−^, NO_3_^−^, and SO_4_^2−^), whereas the quartz filter samples were reserved to determine the content of levoglucosan, organic carbon (OC) and elemental carbon (EC). To eliminate possible contamination, all quartz-fiber filters were pre-fired at 900 °C for 4 h before sampling.

The chemical analysis procedures for soluble ions, OC, EC, levoglucosan, and crustal/metal elements are described in previous publications^45–47^. In summary, each Teflon filter sample (from the Super-SASS device) was shaken with 10 mL deionized water for 60 min, and the extracts were then filtered. Ion chromatography (Dionex ICS-1100, Thermo Scientific) was conducted to analyze the soluble ions in the extracts. The OC and EC concentrations in the quartz filter samples (from the Super-SASS device) were determined with a DRI-2001A carbonaceous aerosol analyzer, following the IMPROVE-A thermo-optical reflectance (TOR) protocol. In addition, a punch (17 mm in diameter) of each quartz filter sample was extracted in 3 mL deionized water for 60 min, and an ion chromatography instrument equipped with a pulsed amperometric detector (PAD) (ICS-5000, Thermo Scientific) was then applied to analyze the content of levoglucosan in the extracts. The Teflon filter samples (from the PQ200 device) were completely dissolved in a mixture of acids in Teflon beakers, i.e., 4 mL HNO_3_ (Merck LTD, 60% ultrapure) and 2 mL HF (Merck LTD, 48% ultrapure). An inductively coupled plasma-mass spectrometry (ICP-MS) instrument (NexION 300X, Perkin Elmer) was employed to determine the concentrations of crustal/metal elements in the digestion solutions. A fraction of each digestion solution was further purified with Sr-Spec resin (100–150 μm, Eichrom Technologies) in a cleanroom, which was in turn used to analyze Pb isotopes via ICP-MS^15^. The National Institute of Standards and Technology SRM 981 Pb standard was used to calibrate the Pb isotopic ratios.

### Back-trajectory cluster analysis

Analysis of the 5-d back-trajectories of air masses was conducted twice per day using the Hybrid Single-Particle Lagrangian Integrated Trajectory (HYSPLIT) model of the National Oceanic and Atmospheric Administration (NOAA) on the sampling days. The meteorological data considered in the model included 6-hourly Global Data Assimilation System (GDAS) archived data with a resolution of 0.5 degrees in longitude and latitude. The end point of the trajectories occurred 200 m above ground level at the Dali station (24.02° N, 120.677° E), Taichung, Taiwan. Trajectory cluster analysis was then conducted to group trajectories into four clusters.

### Positive matrix factorization (PMF) model

We used PMF 5.0 (from the US’s Environmental Protection Administration) model to analyze the pollution source for ambient Pb. The basic principle of the PMF model was described in previous studies^48,49^. The mathematical expression of the PMF model is shown as Eq. (1):

1 $$\chi_{ij} = \sum\limits_{k = 1}^{p} {g_{ik} f_{kj} + e_{ij} }$$

where *x*_*ij*_ is the measured concentration of the *j*th species in the *i*th sample, *p* is a number of factor, *g*_*ik*_ is the contribution of the *k*th pollution source to the *i*th sample, *f*_*kj*_ is the concentration of the *j*th species from the *k*th pollution source, *e*_*ij*_ is the residual for each sample/species.

Because the results are constrained, no sample can have a negative source contribution. PMF allows each data point to be individually weighed. This feature allows the analyst to adjust the influence of each data point, depending on the confidence in the measurement. The PMF solution minimizes the object function Q based on the uncertainties (u) as Eq. (2):

2 $$\mathrm{Q }\left(\mathrm{E}\right)=\sum_{i=1}^{n}\sum_{j=1}^{m}\left[\frac{{X}_{ij}-\sum_{k=1}^{p}{g}_{ij{k}_{ij}}}{{u}_{ij}}\right]$$

Where *uij* is the measured concentration (in μg/m^3^) to the *jth* specie in *ith* sample, *n* is the number of samples, *m* is the number of species. The other parameters are the same as those in Eq. (1). The measured concentrations of PM_2.5_ species were used here and the uncertainty is estimated using Eq. (3). In case with measured concentration of a specific species below method detection limit (MDL), a value of 50% MDL was used as input and the uncertainty is estimated using Eq. (4). We deployed measurements of 26 elements, 3 Pb isotopes (^206^Pb, ^207^Pb, ^208^Pb), 7 ions, OC, EC, and levoglucosan to form the chemical profile in PMF. The novelty here is that the mass concentrations of the three isotopes of lead (^206^Pb, ^207^Pb, and ^208^Pb) in PM_2.5_ samples are measured and treated as 3 independent components in the chemical profile.

3 $$\mathrm{Uncertainty }=\frac{5}{6}\times MDL$$

4 $${\text{Uncertainty }} = \, \left[ {\left( {{\text{Error fraction }} \times {\text{ concentration}}} \right)^{{2}} + \, \left( {0.{5 } + {\text{ MDL}}} \right)^{{2}} } \right]^{{{1}/{2}}}$$

We used Eq. (5) to calculate the contribution of each pollution source to ambient Pb^50^:

5 $$R_{ij} = \sum\limits_{k = 1}^{n} {g_{ik} f_{kj} /X_{ij} }$$

where *R*_*ij*_ is the contribution of the *j*th species in the *i*th sample. The other parameters are the same as those in Eqs. 1 and 2. Then, the chemical profile of each contributing factor resolved by the PMF model contain the variance attributed to the 3 Pb-isotopes, respectively. Given the isotopic contribution, each contributing factor resolved in this study was further characterized by the ratios of Pb-isotopes (i.e. ^206^Pb/^207^Pb and ^208^Pb/^207^Pb).

We also deployed classical Bootstrap procedure to assess the uncertainty for PMF analysis^51,52^. In this study, the number of Bootstrap was assigned at 200 to run uncertainty assessment ^51,53^. If the Bootstrap factor was not correlated with any base factors, the Bootstrap factor was classified as “unmapped”^54^. The detailed results of Bootstrap are described in the Supplementary Fig. 3. The Bootstrap analysis showed that most species, in particular the characteristic constituents for each factor, were contained in the IQR of variance, which suggested that results in base run were robust and representative.

### Two-end-member model

This study used a two end-member model^37^ to calculate the contributions of local source and the East-Asian outflows to the ambient Pb in PM_2.5_ in the CWT following the Eq. (6):

6 $${\text{R }} = {\text{ R}}_{{{\text{EA}}}} \times f_{EA} + {\text{ R}}_{{{\text{LC}}}} \times \left( {{1 } - f_{EA} } \right).$$

where R is the measured Pb isotope ratio (i.e. ^206^Pb/^207^Pb or ^208^Pb/^207^Pb) of a PM_2.5_ sample; R_LC_ and R_EA_ are respectively the Pb isotope ratios of the two end members: the local sources and the East-Asian outflows. In this study, the local end member is characterized by the measurements taken in summertime when Taiwan is isolated from the continental air mass, and the East-Asian end member is characterized by the measurements taken in polluted urban areas in China^41^. *f*_*EA*_ denotes the relative contribution of East-Asian outflows to ambient Pb, and (1—*f*_*EA*_) is the relative contribution of local sources. Then, we separately calculate the relative contributions of local sources and EA outflows to ambient Pb for all sampling sites using ^206^Pb/^207^Pb and ^208^Pb/^207^Pb. Note that the results presented here are based on the analysis of ^208^Pb/^207^Pb because it exhibits a more distinct seasonality than is the ^206^Pb/^207^Pb, and thereby more suitable to this study.

### Statistical analysis

A paired two-tailed t-test and analysis of variance was performed to investigate the differences in the chemical composition of PM_2.5_ between the seasons and sampling sites, respectively. SAS 9.4 (SAS Institute Inc., Cary, NC, USA) statistical software was employed to analyze all data. Statistical significance was defined at *p* < *0.05*.

## Supplementary Information

Supplementary Information.

## Data Availability

The data that support the findings of this study are available from the corresponding author upon reasonable request.
