# Supplementary material for: Isotopic signatures and source apportionment of Pb in ambient PM2.5
Source: Sci Rep. 2022 Mar 14;12:4343. doi: 10.1038/s41598-022-08096-1 (PMC8921186; doi:10.1038/s41598-022-08096-1)
Supplement: Supplementary file 1 — Supplementary Information. [file 41598_2022_8096_MOESM1_ESM.docx]

Isotopic signatures and source apportionment of Pb in ambient PM_2.5_

**Supplementary Information**

Jung *et al.*, submitted to Scientific Reports

**Table 1:** Detailed information on the sampling campaigns conducted during the intensive field investigation from 2016–2018

| **Site Category** | **Site ID** | **Latitude and Longitude** | **Sample Size** | **Sampling period**  **(mm/yy)** |
| --- | --- | --- | --- | --- |
| Urban | Changhua  (CH) | 24°03’57’’ N, 120°32’29’’ E | 36 | 11/2016, 2/2017, 8/2017, 3/2018, and 8/2018 |
|  | Douliu(DL) | 23°42’42’’ N, 120°32’41’’ E | 23 | 11/2016, 2/2017, and 8/2017 |
|  | Dali(DA) | 24°05’58’’ N, 120°40’39’’ E | 14 | 3/2018 and 7/2018 |
|  | Fengyuan(FY) | 24°15’23’’ N, 120°44’30’’ E | 14 | 3/2018 and 7/2018 |
|  | Chiayi(CY) | 23°27’46’’ N, 120°26’27’’ E | 23 | 11/2016, 2/2017, and 8/2017 |
| Rural | Erlin(EL) | 23°55’30’’ N, 120°24’34’’ E | 23 | 11/2016, 2/2017, and 8/2017 |
|  | Shalu(SL) | 24°13’32’’ N, 120°34’07’’ E | 14 | 3/2018 and 7/2018 |
|  | Xianxi(XX) | 24°07’54’’ N, 120°28’08’’ E | 14 | 3/2018 and 7/2018 |
|  | Xiangang(XG) | 23°33’17’’ N, 120°20’43’’ E | 22 | 11/2016, 2/2017, and 8/2017 |
|  | Zhushan(ZS) | 23°45’23’’ N, 120°40’38’’ E | 23 | 11/2016, 2/2017, and 8/2017 |
| Industrial | Mailiao(ML) | 23°45’12’’ N, 120°15’06’’ E | 22 | 11/2016, 2/2017, and 8/2017 |
|  | Lunbei(LB) | 23°45’27’’ N, 120°20’55’’ E | 23 | 11/2016, 2/2017, and 8/2017 |
|  | Taixi(TX) | 23°43’03’’ N, 120°12’10’’ E | 23 | 11/2016, 2/2017, and 8/2017 |

**Table 2:** Summary of the estimates of the local and continental contributions to the ambient Pb level in PM_2.5_ during the seasons of East Asian outflows in central-western Taiwan

| **Sampling site** | **Mass concentration of Pb in PM_2.5_ (ng/m^3^)** | **Contribution of EA outflows**  **(ng Pb/m^3^)** | **Contribution of local sources**  **(ng Pb/m^3^)** |
| --- | --- | --- | --- |
| Changhua (CH) | 12.7 | 5.3 (42 %) | 7.4 (58 %) |
| Douliu (DL) | 12.4 | 4.2 (34 %) | 8.2 (66 %) |
| Dali (DA) | 7.5 | 3.8 (50 %) | 3.7 (50 %) |
| Fengyuan (FY) | 4.8 | 2.8 (58 %) | 2.0 (42 %) |
| Chiayi (CY) | 21.1 | 1.4 (7 %) | 19.7 (93 %) |
| Erlin (EL) | 11.9 | 5.1 (43 %) | 6.8 (57 %) |
| Shalu (SL) | 4.7 | 0.8 (16 %) | 3.9 (84 %) |
| Xianxi (XX) | 9.6 | 2.9 (30 %) | 6.7 (70 %) |
| Xingang (XG) | 10.5 | 3.6 (34 %) | 6.9 (66 %) |
| Zhushan (ZS) | 9.3 | 4.1 (44 %) | 5.2 (56 %) |
| Mailiao (ML) | 9.8 | 2.2 (22 %) | 7.6 (78 %) |
| Lunbei (LB) | 12.2 | 5.4 (45 %) | 6.8 (55 %) |
| Taixi (TX) | 8.6 | 5.4 (63 %) | 3.2 (37 %) |
| Overall | 10.4 | 3.6 (35 %) | 6.8 (65 %) |

**Table 3: All pollution source factors for Pb in PM_2.5_ and Pb isotopic composition of the respective source factors resolved by the PMF model**

| PMF factor  (attribution) | Contribution, %* | ^206^Pb/^207^Pb | ^208^Pb/^207^Pb | Characteristic constituents^#^ |
| --- | --- | --- | --- | --- |
| Factor 1 | 12 ± 5  (S: 12 ± 6; W: 12 ± 7) | 1.146 ± 0.005 | 2.418 ± 0.009 | Zn (51%), Cu (31%), Mn (23%), Pb (12%)^1^ |
| Factor 2 | 11 ± 4  (S: 16 ± 8; W: 8 ± 3) | 1.148 ± 0.009 | 2.292 ± 0.008 | Nd (56%), Ce (51%), Ti (48%), La (39%), Sr (26%)^2, 3, 4^ |
| Factor 3 | 49 ± 12 %  (S: 35 ± 16; W: 57 ± 24) | 1.159 ± 0.004 | 2.467 ± 0.006 | Cd (57%), Pb (52%), As (40%), Se (32%)^5^ |
| Factor 4 | 10 ± 5  (S: 21 ± 8; W: 6 ± 2) | 1.126 ± 0.004 | 2.278 ± 0.005 | V (65%), Ni (43%)^6, 7^ |
| Factor 5 | 6 ± 6 %  (S: 7 ± 3; W: 3 ± 3) | 1.134 ± 0.004 | 2.442 ± 0.010 | Sulfate (56%), OC (51%), Cr (43%), EC (40%), NH_4_^+^ (34%), Ca (27%) |
| Factor 6 | 7 ± 3  (S: 7 ± 4; W: 7 ± 4) | 1.125 ± 0.017 | 2.517 ± 0.017 | levoglucosan (69%), K^+^ (20%)^8, 9^ |
| Factor 7 | 7 ± 2 %  (S: 2 ± 2; W: 7 ± 2) | 1.158 ± 0.010 | 2.430 ± 0.009 | NO_3_^-^ (66%), NH_4_^+^ (33%) |
| Factor 8 | < 1 | - | - | Cl^-^ (73%), Mg^+2^ (51%), Na^+^ (39%)^10^ |

*Averages for summer (S) and winter (W) campaigns are listed in parentheses. ^#^ The percentage of variations in the characteristic elements attributed to each PMF factor is listed in parentheses. **Factor 1:** Traffic emission; **Factor 2:** Petrol industry; **Factor 3:** Coal-fired facilities; **Factor 4:** Oil-fired facilities; **Factor 5:** Sulfate and mix industrial pollution; **Factor 6:** Biomass burning; **Factor 7:** Secondary aerosol; **Factor 8:** Sea salt.

**
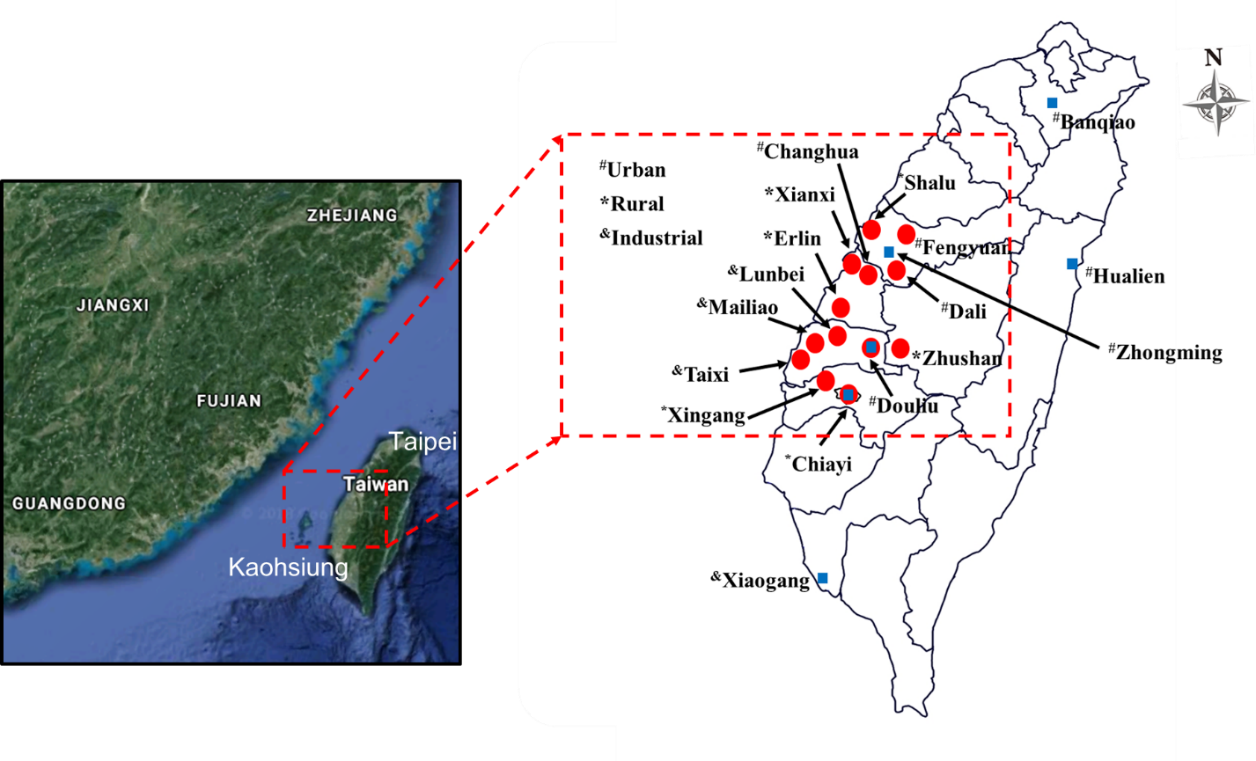
**

**Figure 1:** Geographic locations of Taiwan in East Asia and the sampling sites in central-western Taiwan. The red circles indicate the sites deployed during the intensive investigation in this study. The blue squares indicate the sites of the Taiwan EPA PM_2.5_ speciation network. Note that the Chiayi and Douliu sites are deployed under both sampling programs.


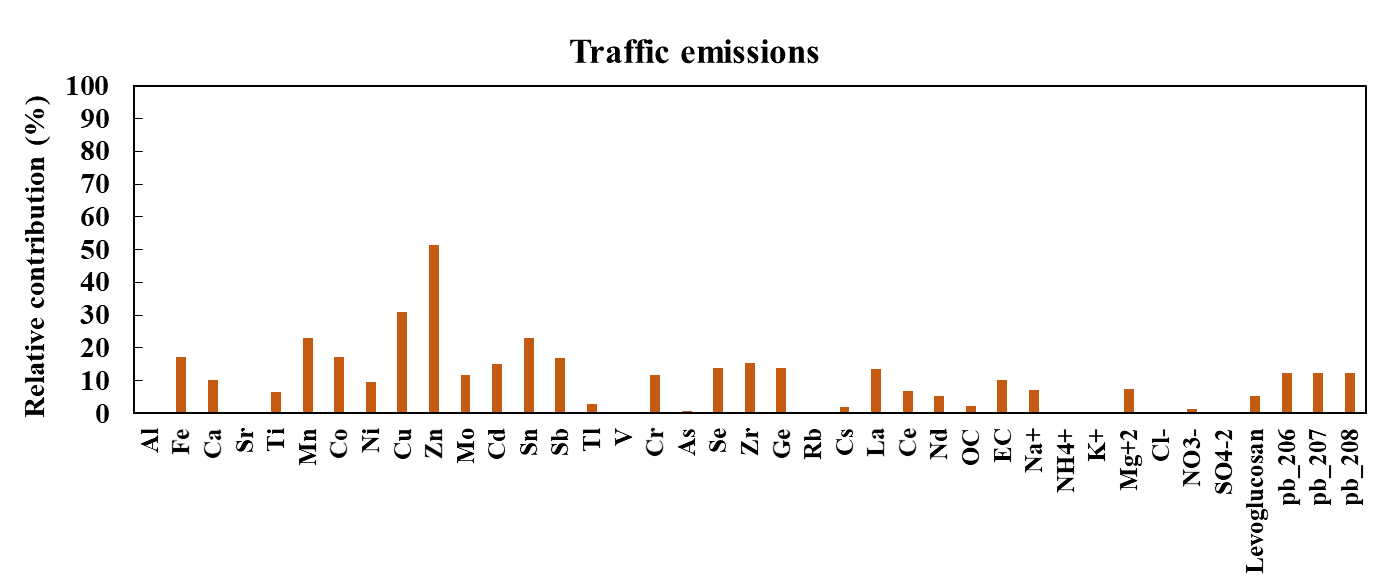


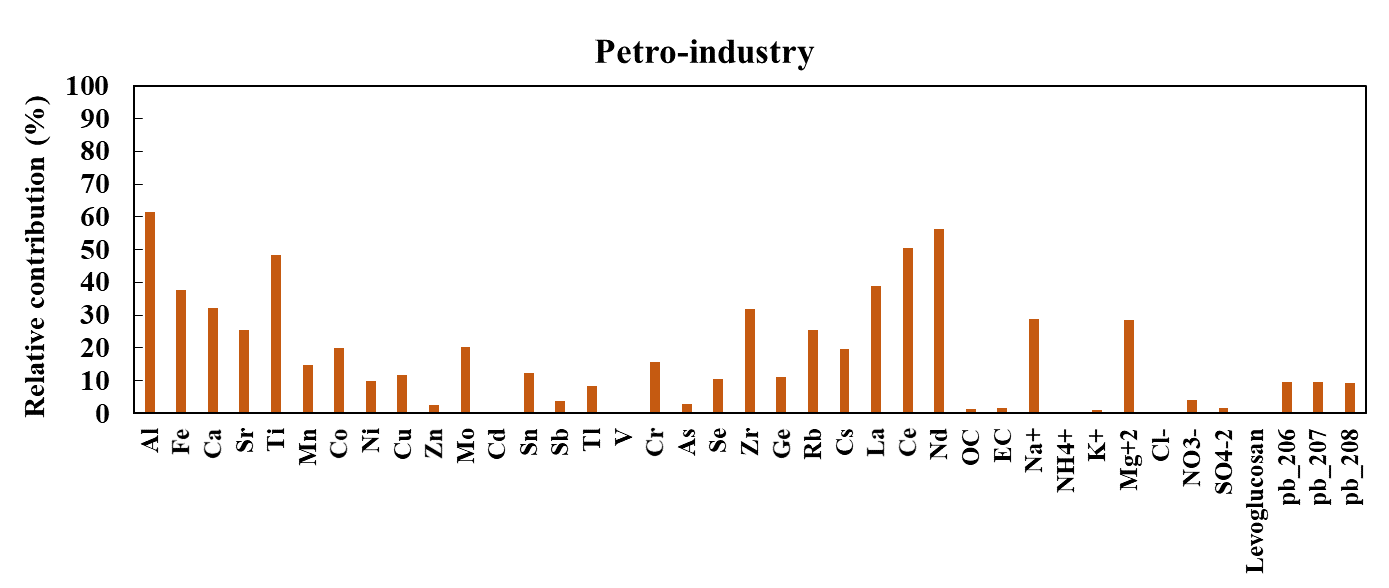


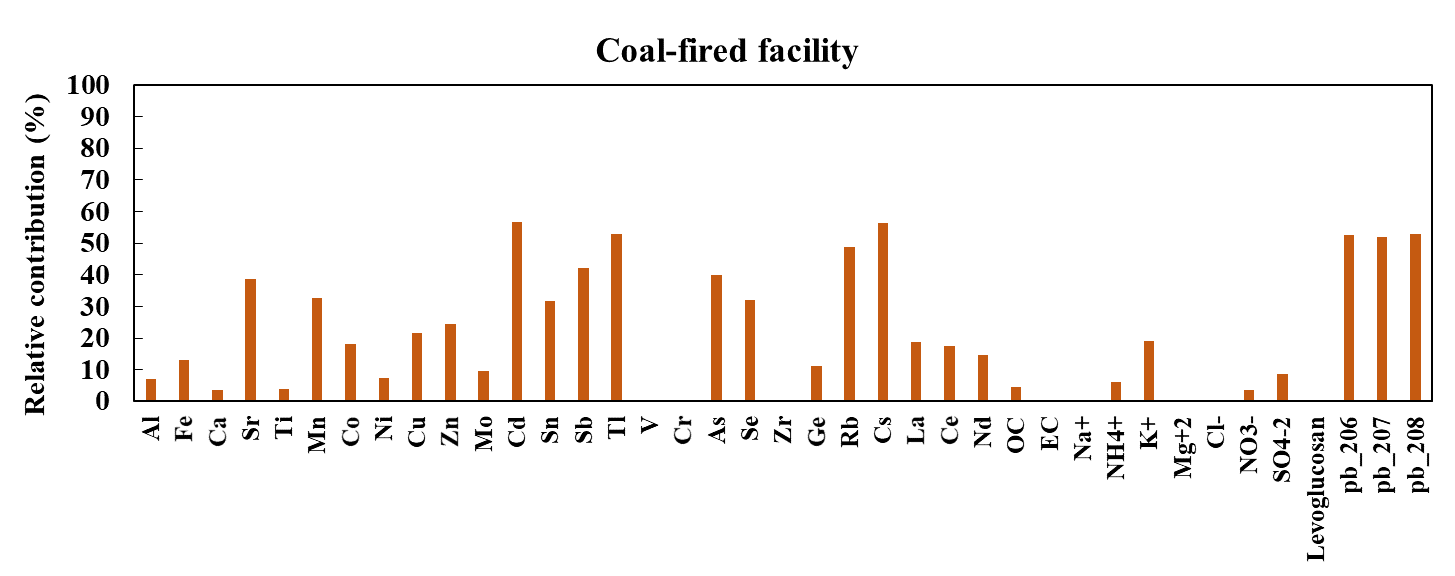


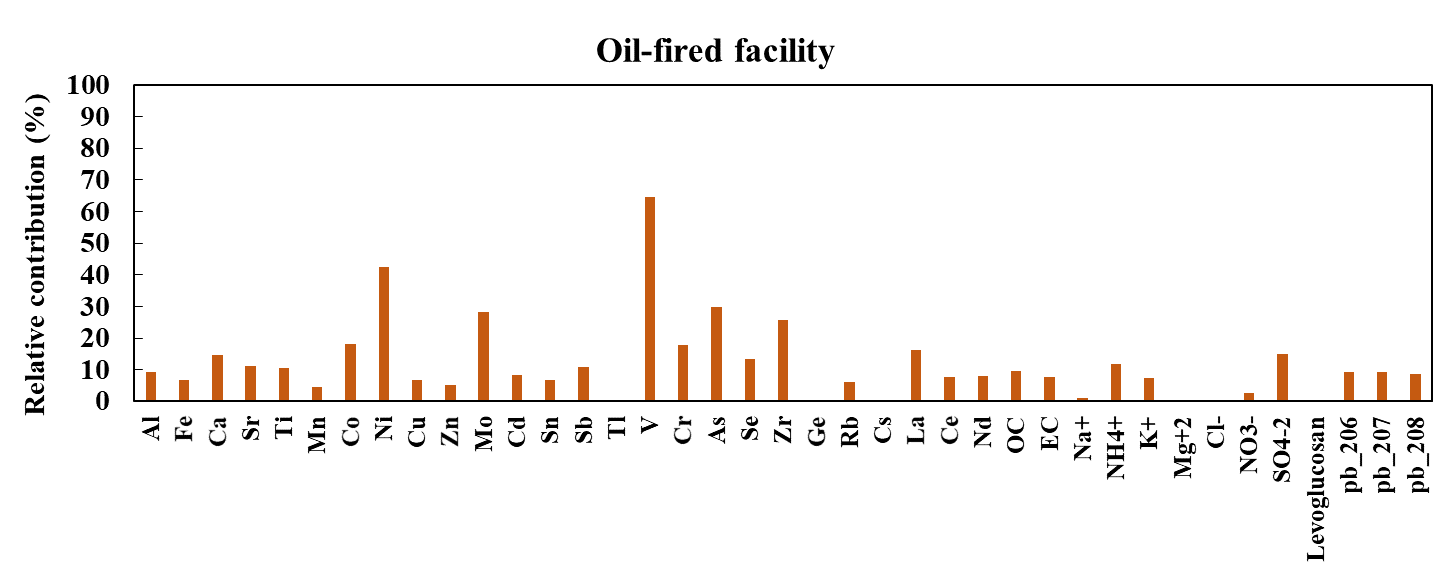


**Figure 2:** Chemical profiles of the specific source factors resolved by the PMF model


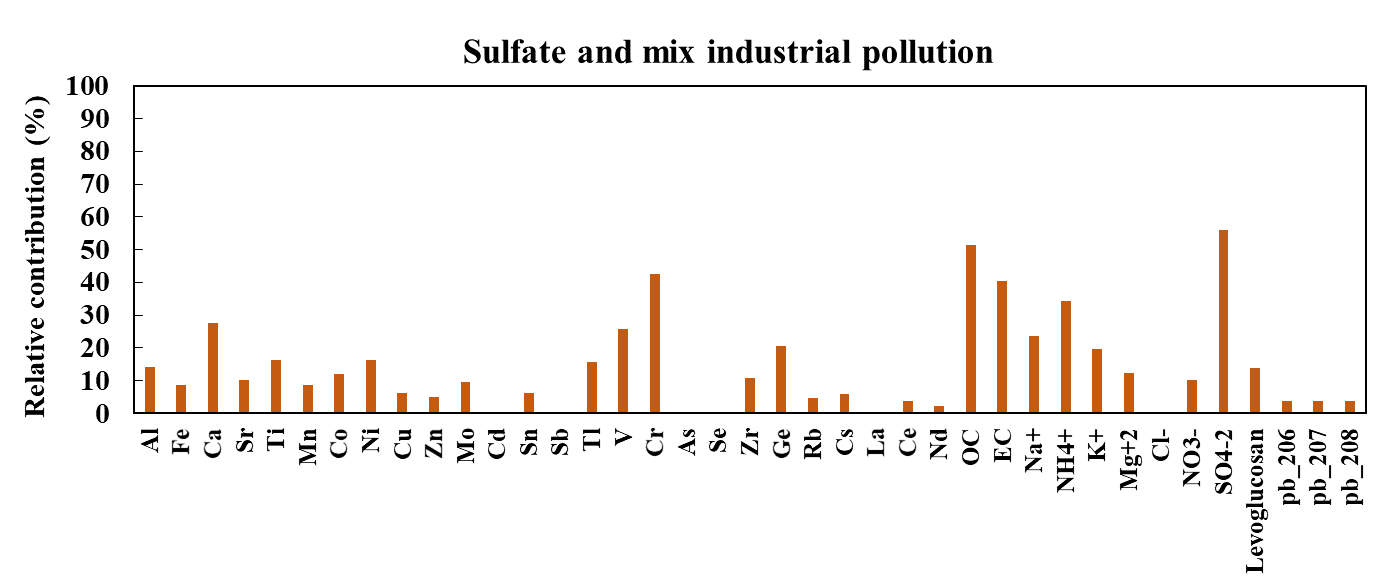


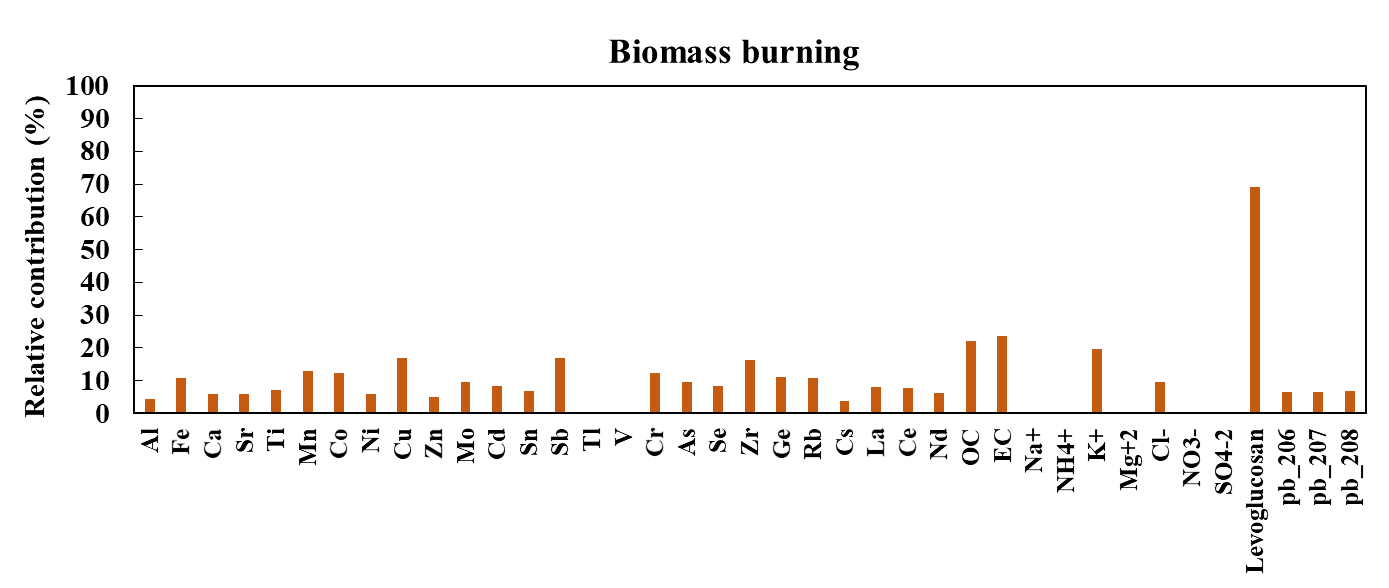


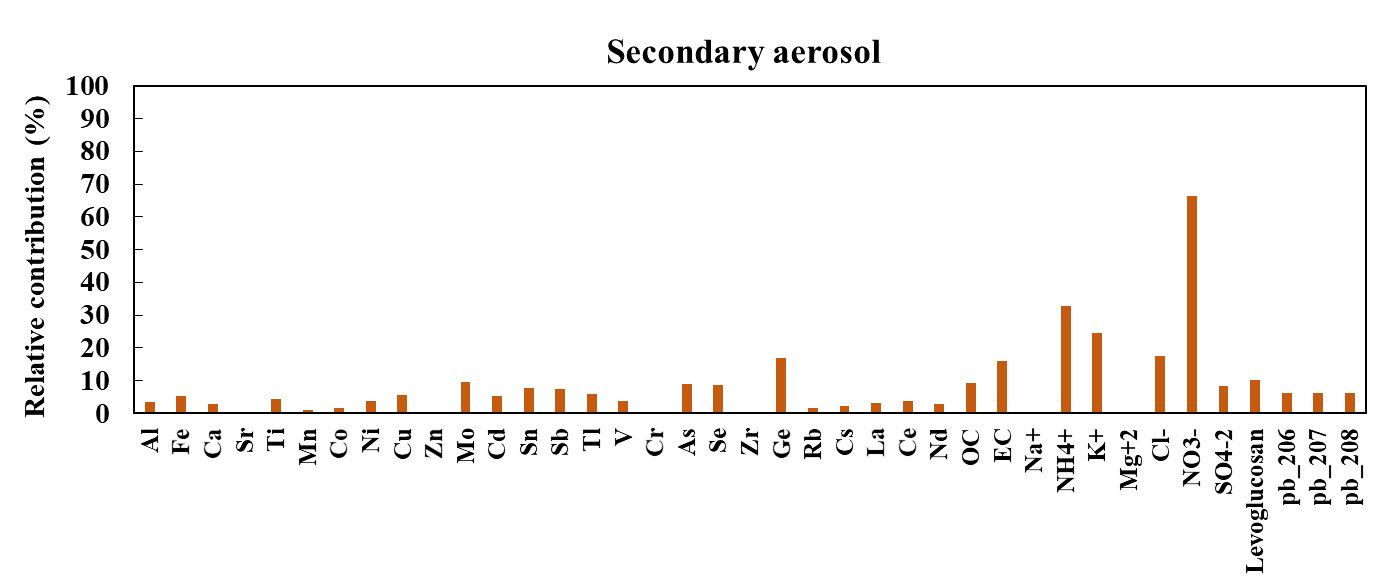


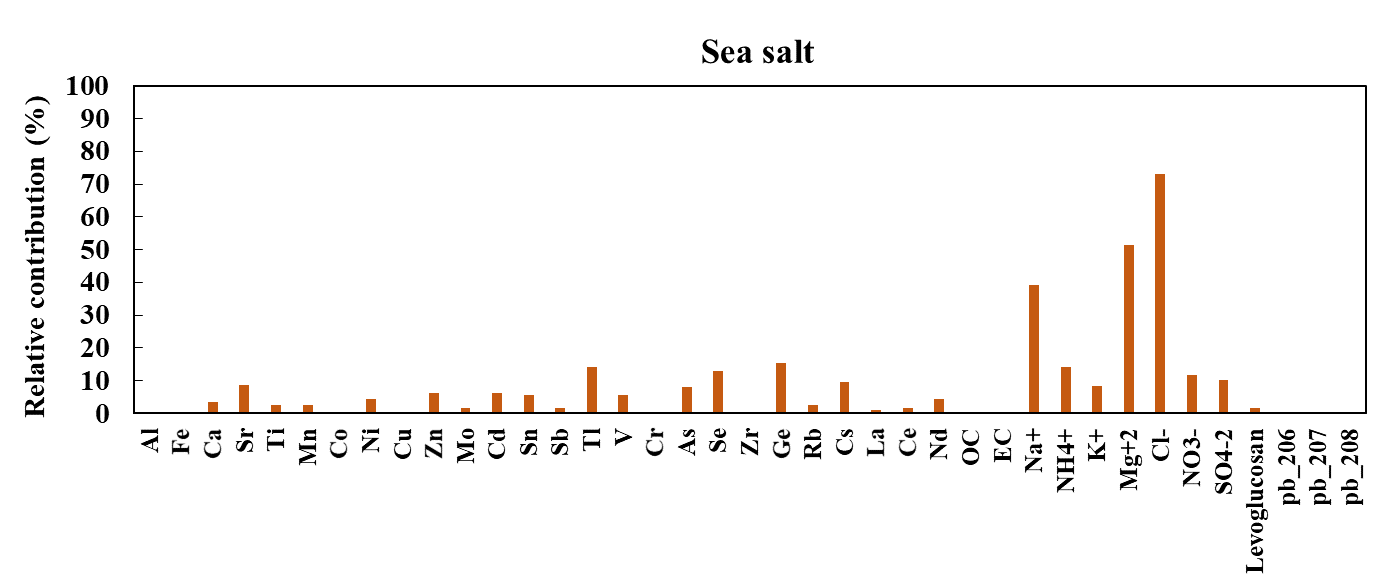


**Figure 2:** Chemical profiles of the specific source factors resolved by the PMF model.

**
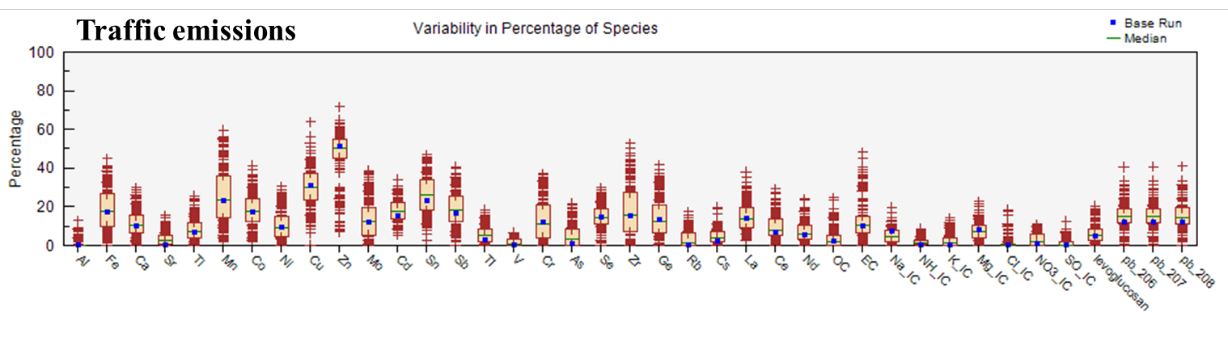
**

**
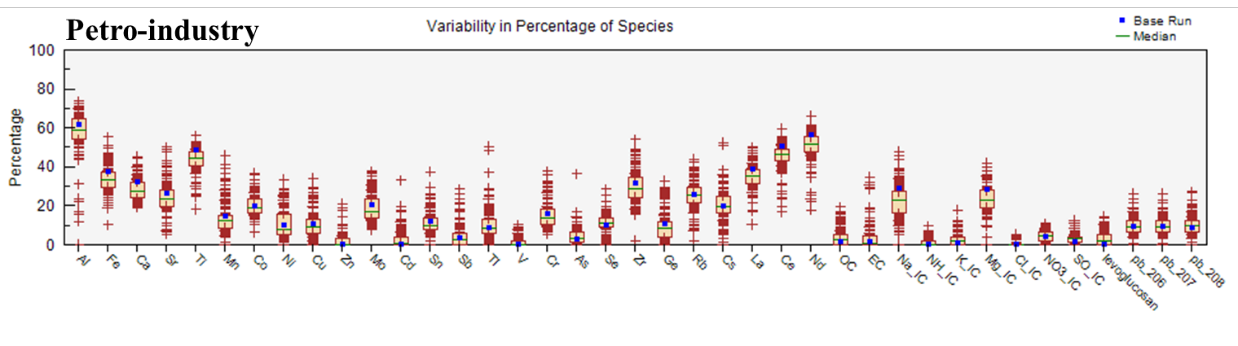
**

**
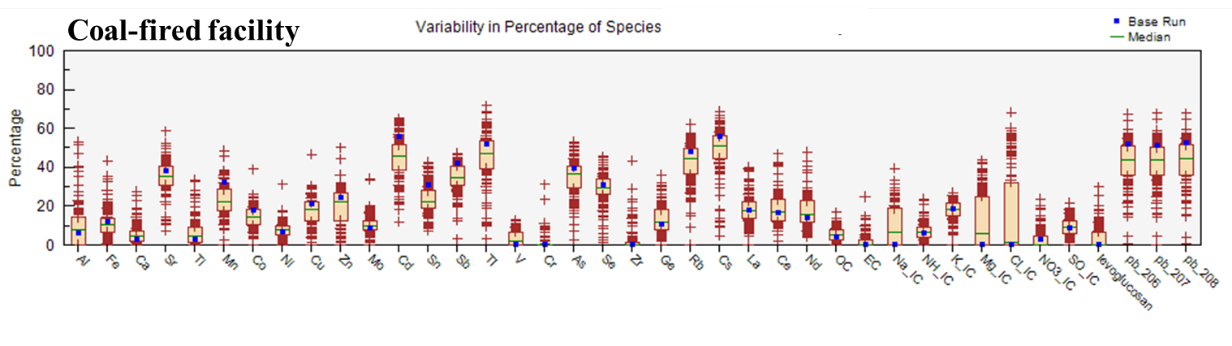
**

**
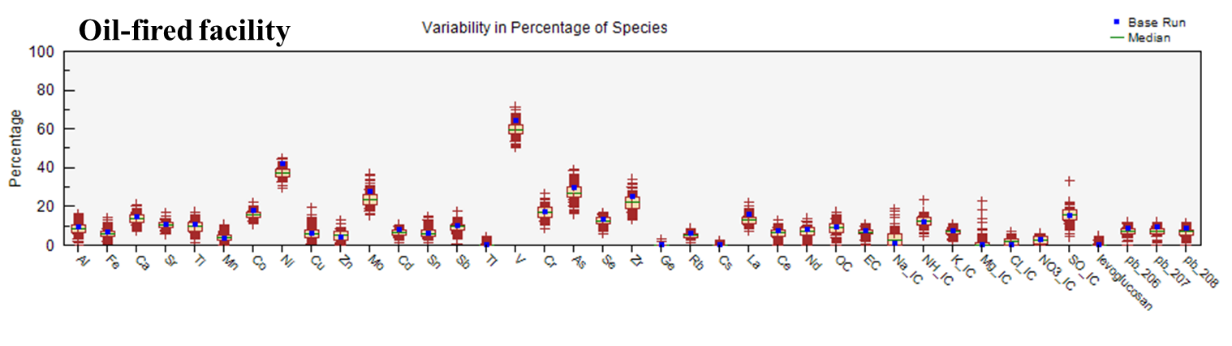
**

**Figure 3:** Output of bootstrap run for each factor from PMF. The base run is shown as a blue box-plot for reference. The box-plots show the interquartile range (IQR, 25^th^-75^th^ percentile) of the Bootstrap run. The horizontal green line and red cross line represent the median and the values outside the IQR, respectively.

**
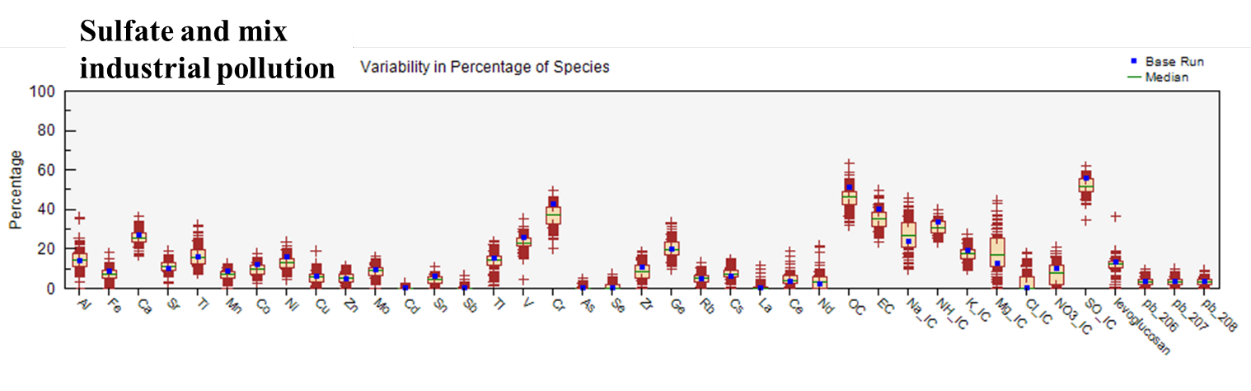
**


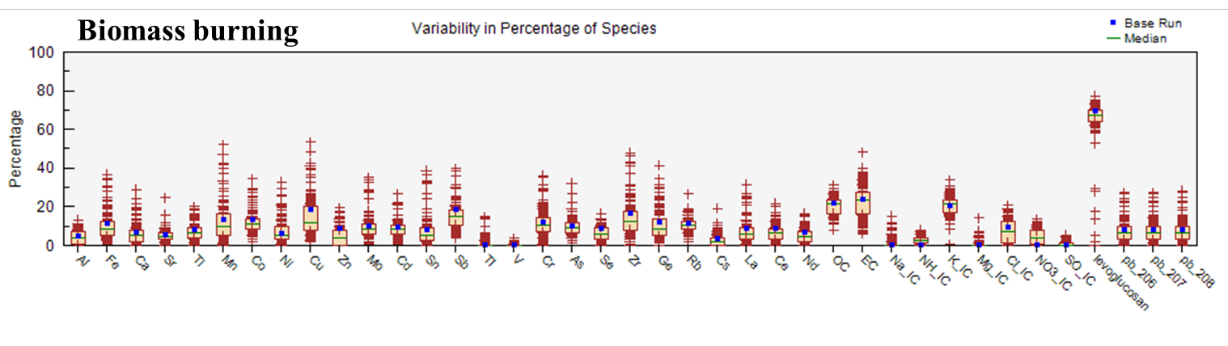


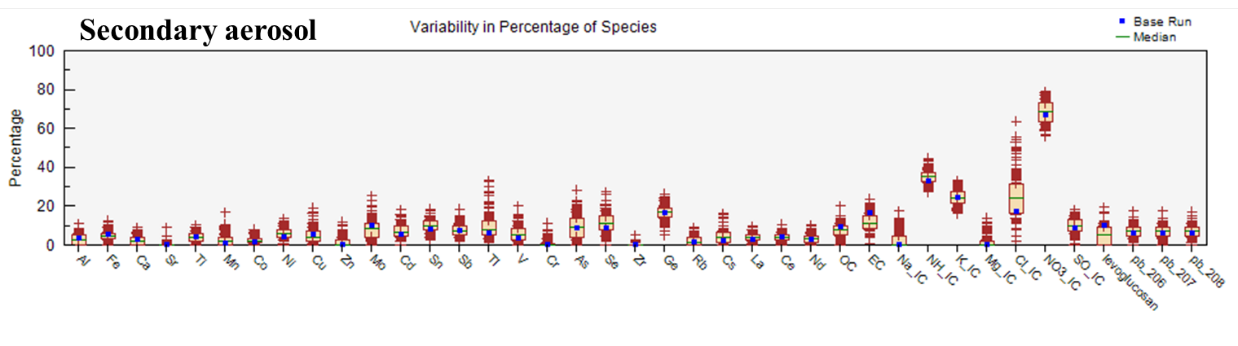


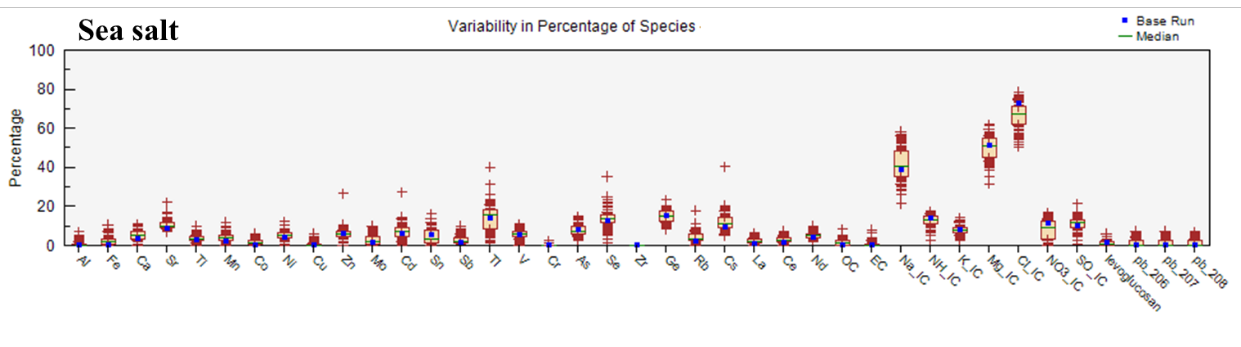


**Figure 3:** Output of bootstrap run for each factor from PMF. The base run is shown as a blue box-plot for reference. The box-plots show the interquartile range (IQR, 25^th^-75^th^ percentile) of the Bootstrap run. The horizontal green line and red cross line represent the median and the values outside the IQR, respectively.


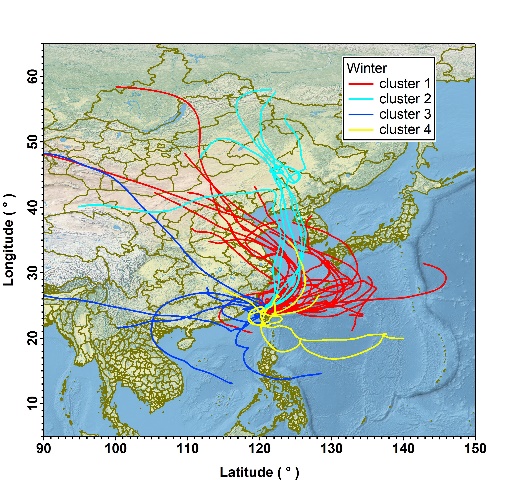

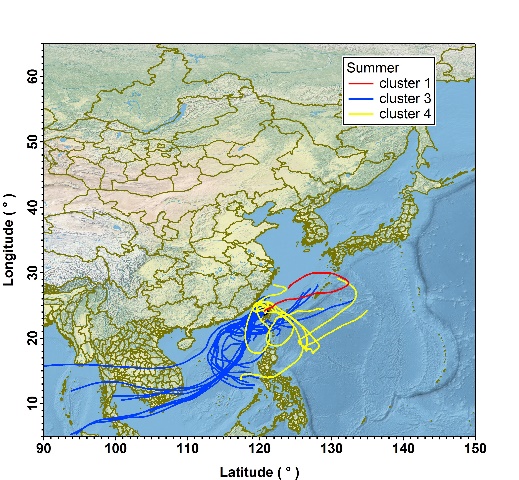


**Figure 4:** Cluster classification of the 5-d back-trajectories during the measurement period. The data for the back-trajectory plot overlaid on the map are obtained from the NOAA HYSPLIT model. Clusters 1 and 2 are air masses associated with East Asian continental outflows induced by high-pressure systems during the autumn to spring seasons. The air masses of both clusters 1 and 2 originate in inland regions of the Asian continent, but the movement of the cluster 2 air masses is faster and originates from higher altitudes. The air masses of clusters 3 and 4 are associated with low-pressure systems during the summer season and occasionally occur during the spring season, which induces the transport of air masses from Pacific areas to the Taiwan region. The digital map is generated with Igor Pro 6.37 (© WaveMetrics)

**References**

1. Lin Y-C*, et al.* Characteristics of trace metals in traffic-derived particles in Hsuehshan Tunnel, Taiwan: size distribution, potential source, and fingerprinting metal ratio. *Atmospheric Chemistry and Physics* **15**, 4117-4130 (2015).

2. Chow JC*, et al.* Source profiles for industrial, mobile, and area sources in the Big Bend Regional Aerosol Visibility and Observational study. *Chemosphere* **54**, 185-208 (2004).

3. Kulkarni P, Chellam S, Fraser MP. Tracking petroleum refinery emission events using lanthanum and lanthanides as elemental markers for PM2. 5. *Environmental science & technology* **41**, 6748-6754 (2007).

4. Moreno T, Querol X, Alastuey A, Gibbons W. Identification of FCC refinery atmospheric pollution events using lanthanoid-and vanadium-bearing aerosols. *Atmospheric Environment* **42**, 7851-7861 (2008).

5. Okuda T*, et al.* Trends in hazardous trace metal concentrations in aerosols collected in Beijing, China from 2001 to 2006. *Chemosphere* **72**, 917-924 (2008).

6. Cheng M-T, Chio C-P, Huang C-Y, Chen J-M, Wang C-F, Kuo C-Y. Chemical compositions of fine particulates emitted from oil-fired boilers. *Journal of Environmental Engineering and Management* **18**, 355-362 (2008).

7. Querol X*, et al.* Source origin of trace elements in PM from regional background, urban and industrial sites of Spain. *Atmospheric Environment* **41**, 7219-7231 (2007).

8. Cheng M-T, Horng C-L, Su Y-R, Lin L-K, Lin Y-C, Chou CC-K. Particulate matter characteristics during agricultural waste burning in Taichung City, Taiwan. *Journal of hazardous materials* **165**, 187-192 (2009).

9. Simoneit BR*, et al.* Levoglucosan, a tracer for cellulose in biomass burning and atmospheric particles. *Atmospheric Environment* **33**, 173-182 (1999).

10. Rahn KA. A graphical technique for determining major components in a mixed aerosol. I. Descriptive aspects. *Atmospheric Environment* **33**, 1441-1455 (1999).
